# The Institutional Worker

**Published:** 1912-09-14

**Authors:** 


### INSTITUTIONAL WORKER

**Being a Special Supplement to "The Hospital"**

### Editor's Notices.

**Contributions:**

Contributions should be written, or preferably typed, on one side of the paper only, and all articles sent in are accepted upon the distinct understanding that they are forwarded to THE HOSPITAL only.

The Editor cannot undertake to return MSS. not used, but when a stamped directed envelope is enclosed the MSS. may be returned if a special request is made.

Accepted articles and paragraphs of news will be paid for after publication at the scale rate.

**Address:**

To prevent delay all contributions and letters on editorial business must be addressed exclusively to the Editor, "The Hospital," 29 Southampton Street, Strand, London, W.C. It is important that this regulation shall be strictly observed.

**Correspondence:**

Correspondence on all subjects is invited. The name and address of correspondents must be given as a guarantee of good faith, but not necessarily for publication.

**Special Articles:**

Special articles are invited, and questions, inquiries, and paragraphs upon all matters relating to the work, administration, and management of general, special, mental, fever, cottage and convalescent hospitals, sanatoria, homes, institutions, societies and organisations for the treatment or care of the sick, injured and dependants of all classes. Every matter affecting the interests and welfare of the staff of all grades working in these institutions will receive special consideration.

**Administrative Medicine, Research and Items of News:**

Special terms will be paid for approved articles on subjects in this wide field by experts who have made some section of it their special study and interest. We wish to give prominence to every movement tending to advance accuracy of diagnosis, efficiency in treatment, and the development of modern methods to eradicate disease and promote the welfare of the suffering.

**A Bureau of Information:**

We invite inquiries and applications for information and help in providing for that numerous class of sufferers who require special care or house-room which they cannot obtain in their own homes or secure for themselves. **We cannot, however, prescribe, or recommend practitioners.**

**Books for Review:**

Publishers are particularly requested to send advance proofs of any new books of importance whenever possible, as the Editor has made arrangements to publish immediate reviews on a new plan.

**Photographs, Plans, Blocks, and Illustrations:**

It is requested that wherever possible MSS. may be accompanied by illustrations in any of the above forms. The name of the sender and of the article to which it belongs should be written on the back of each photograph, drawing, or block for purposes of identification.

**Local Papers:**

Newspapers containing reports or news paragraphs **should be marked** and addressed to the Sub-Editor.

**The Coupon System:**

A coupon will be found at the bottom of the third inside page of the cover each week. This coupon must be attached to every question or inquiry to which an answer is desired in THE HOSPITAL.

### Suggestions Needed.

As it is our purpose to make the Bureau of Information

**A World-wide Influence and Service,**

a cordial invitation is given to our readers in all parts of the globe to send suggestions for the extension of the work of the Bureau as may be calculated to render it of the greatest possible utility to English persons resident or travelling abroad.

The many communications received daily from Institutional and Social Workers for information and practical help prove that the Bureau is meeting a definite and long-felt want, and with the assistance of our readers it is our intention to widen the scope and increase the beneficial work of this new department of THE HOSPITAL.

All communications should be addressed to THE HOSPITAL Bureau of Information, THE HOSPITAL Building, 28 and 29 Southampton Street, Strand, London, W.C.

### Manager's Notices.

**All letters on business matters must be addressed to The Manager, "The Hospital," 28 and 29 Southampton Street, London, W.C.**

**Advertisements:**

To ensure insertion, all advertisements for THE HOSPITAL must reach the Manager not later than Wednesday at noon in each week. For scale of charges see pages v. and 2.

**Subscriptions,** which may commence at any time, are payable in advance. Cheques and Post-Office Orders should be crossed London County and Westminster Bank, Covent Garden Branch, and made payable to the Manager of THE HOSPITAL.

RATES OF SUBSCRIPTION (including Postage).

| *United Kingdom.* | | *Foreign and Colonial.* | |
|---|---|---|---|
| Three Months ... ... | 2s. 0d. | Three Months ... ... | 2s. 6d. |
| Six Months ... ... | 4s. 0d. | Six Months ... ... | 5s. 0d. |
| Twelve Months ... ... | 6s. 6d. | Twelve Months ... ... | 8s. 8d. |

*SUBSCRIPTION FORM.*

*Please send me* THE HOSPITAL *for*..................*months, commencing with your issue of*.............................., *for which please find enclosed*............................

*Name*..................................................

*Address* ..................................................

..................................................

*Date*....................................

*To* .................................. *Newsagent.*

**Remittances:**

**It is especially requested that remittances be made by Cheque or Postal Order, and in halfpenny stamps only when the amount is under sixpence.**

### The Scientific Press Publications

### INSTITUTIONAL WORKS.

### HOSPITALS AND ASYLUMS OF THE WORLD.

**By SIR HENRY BURDETT, K.C.B., K.C.V.O.**

In Four Volumes, with Separate Portfolio containing several hundred Plans.

**Vols. I. and II. (ASYLUMS)** ... **Price £6 15s.** | **Vols. III. and IV. (HOSPITALS) and the Portfolio of Plans** ... **Price £9**

**The Portfolio of Plans** (20 in. × 14 in., drawn to uniform scale), **Price £4 14 6**

**The Complete Work** ... ... **£12 12s.**

This magnificent work forms a Complete Survey of the Origin, History, Construction, Administration and Management of the Hospitals and Asylums of the World, with detailed information concerning the principal Institutions.

*Only a few copies remain unsold.*

### LEDGERS, ACCOUNT BOOKS, STOCK BOOKS of all kinds for large and small Institutions.

These are supplied already ruled and printed, or in accordance with the special requirements of institution managers.

### ANNUAL REPORTS AND PROSPECTUSES.

THE SCIENTIFIC PRESS are prepared to undertake the production of the Annual Reports and Prospectuses of every class of Institution, and to supply printed or typewritten copies of testimonials for the officials. Professional Cards.

### TEMPERATURE CHARTS.

**MORNING, EVENING, FOUR-HOUR, MATERNITY.**

Size 10 inches by 8 inches 12 3d. 25 6d. 50 9d. 100 1/4 | 250 3/3 500 6/6 1,000 12/6

Postage extra.

All Orders for 250 and over are sent Carriage Paid.

### CASE PAPERS

**For Day and Night Nurse's Reports.** Bound & unbound.

### DISTRICT NURSING ASSOCIATIONS'

**Case, Visit, Time, and Loan and Account Books.**

### INSTITUTIONAL LIBRARY.

**THE SCIENTIFIC PRESS will procure literature, professional and otherwise, for the formation of and addition to Institutional Libraries. It is economy of time and expenditure to utilise one source of supply.**

**THE SCIENTIFIC PRESS, LTD.,**

**28 and 29 Southampton Street, Strand, London, W.C.**

SEND FOR COMPLETE CATALOGUE.

